# The living dinosaur: accomplishments and challenges of reconstructing dinosaur physiology

**DOI:** 10.1098/rsbl.2025.0126

**Published:** 2025-05-29

**Authors:** Stephanie L. Baumgart, Clinton A. Grand Pré, Jason M. Bourke, Emma R. Schachner

**Affiliations:** ^1^ Department of Physiological Sciences, University of Florida College of Veterinary Medicine, Gainesville, FL, USA; ^2^ Department of Anatomical Sciences, Stony Brook University Renaissance School of Medicine, Stony Brook, NY, USA; ^3^ Department of Biomedical and Anatomical Sciences, New York Institute of Technology College of Osteopathic Medicine at Arkansas State University, Jonesboro, AR, USA

**Keywords:** Dinosauria, physiology, metabolism, thermoregulation, respiration, biological correlates

## Abstract

The drive to determine the physiology of non-avian dinosaurs has produced several novel methodologies. In this review, we survey the current state of the field and evaluate the latest reconstructions of non-avian dinosaurian metabolism, thermoregulation, respiratory biology, and the cardiovascular and digestive systems. Most inferences of dinosaur physiology are based on correlations that assume tightly linked relationships between physiology and anatomy. Such causal links must first be well established, tested and functionally validated across a phylogenetically broad range of extant taxa before they can be applied to extinct forms. We offer some basic guidelines for conducting evidence-based, robust reconstructions of palaeophysiology that stay within the confines of both empirical studies and the fossil record.

## 1. Introduction

Throughout the past 200 years, palaeontologists have recovered thousands of dinosaur fossils (see electronic supplementary material, [1]). These discoveries vastly increased our knowledge of how dinosaurs appeared in life, yet questions of dinosaur physiology have historically remained largely speculative (e.g. [2,3]). Only with technological advancements of the late twentieth/early twenty-first century can we now quantitatively test hypotheses of dinosaur palaeophysiology [4–9]. Using innovative methods, palaeontologists have mined the fossil record for clues regarding myriad physiological processes [6,10–16]. These methods offer glimpses into how these extinct animals lived, but method limitations constrain the extent to which solid inferences of palaeophysiology can be made. It is critical for physiological interpretations of extinct animals to be grounded within a systematic methodology of rigorously validated studies. Here, we review some of the latest methods for studying palaeophysiology. As the scope of physiology is vast, with several aspects of dinosaur biology covered by other contributions in this volume [17–20], we limited our discussion to dinosaur metabolism, thermoregulation and three large biological systems (respiratory, cardiovascular and digestive). We end our review with some suggested guidelines for strengthening future palaeophysiological hypotheses.

## 2. Methods in dinosaur thermophysiology

Life has evolved multiple solutions for maintaining thermal/metabolic autonomy over a range of environmental temperatures, running a continuum from near total reliance on external methods of thermoregulation (ectothermy) to near total reliance on internal methods (endothermy). Several palaeontological methods have been developed to search for thermophysiological proxies, allowing for better reconstructions of dinosaur metabolism.

### (a) Skeletochronology

We briefly touch on dinosaur skeletochronology as most studies focus on growth rates, which are covered in a separate contribution to this volume [17]. Thin sections of bone yield information on the rate of bone deposition in life, and by extrapolation, how fast the whole animal grew [21]. Bone deposition rates have been used to infer the underlying metabolism of extinct animals. Slow bone deposition is associated with bradymetabolic ectotherms, whereas fast deposition is associated with tachymetabolic, obligate endotherms (i.e. mammals and birds) [21–23]. Initial studies of non-avian dinosaur bone revealed bone textures commonly found in mammals and birds, leading to interpretations of obligate endothermy in dinosaurs [24,25]. In contrast, initial discoveries of lines of arrested growth (LAGs) in dinosaur bones indicated a seasonal change in growth rate that led to interpretations of a more bradymetabolic physiology [26,27].

### (b) Clumped isotope palaeothermometry

Temperature-dependent changes in oxygen isotope ratios (^18^O : ^16^O) captured in bone offer potential as a proxy for predicting body temperatures of extinct animals (palaeothermometry) [28–31]. When applied to non-avian dinosaurs, these methods revealed high and stable body temperatures, supporting inferences of obligate endothermy [28–31]. However, reviews of these methods found weak statistical support for temperature inferences in extinct animals [32,33]. A newer method called ‘clumped isotope’ palaeothermometry [11] has been adopted. It exploits tendencies for certain carbon isotopes (^13^C) to bind or ‘clump’ with oxygen (^18^O) at a given temperature. This approach has proven more resilient to the vagaries of fossilization [34].

Clumped isotope palaeothermometry studies of sauropods recovered estimated body temperatures of 36−38°C [11], falling within the range of calculated core body temperatures for ‘sauropod-sized’ crocodylians and birds [35]. When compared to estimated environmental temperatures from the same localities, these data suggested that sauropods actively thermoregulated [11]. Follow-up studies applying this method to fossilized eggshells estimated core body temperatures of female non-avian dinosaurs at the time of egg laying [36–39]. Estimated core temperatures were considerably variable for both sauropods and theropods (29−46°C [37]; 28−44°C [38]). Despite this wide temperature variation, all non-avian dinosaur values were higher than estimated environmental temperatures from each locality and indicative of some form of thermoregulation in these dinosaurs regardless of their thermophysiology. Palaeothermometry offers a tangible method for ‘taking the temperature’ of extinct animals. These estimates provide a range of values that approximate the animal’s core body temperature, but they cannot describe how the animal achieved that temperature.

### (c) Advanced lipoxidation end-products

A recently developed method may be able to capture the fossil trace of cellular activity using advanced lipoxidation end-product (ALE) signals. This method uses the ratio of cross-links between preserved organic molecules and their more geologically stable variants [10]. ALEs are a by-product of reactive oxygen species (ROS) formed during ATP synthesis. Researchers have hypothesized that this causal relationship can be used to determine the thermophysiology of an organism [10]. Higher ALE cross-link ratios should be present in obligate endotherms as they produce more ROS. This method was applied to extinct amniotes based on an extant dataset correlating resting metabolic rate (RMR) with ALEs. Results categorized most non-avian dinosaurs, pterosaurs and plesiosaurs as obligate endotherms, whereas ornithischians and *Pteranodon* appeared to be secondarily ectothermic [10].

## 3. Thermoregulation

Whereas dinosaur thermophysiology remains ambiguous, more focused studies looking at thermoregulatory structures and systems offer insights into thermoregulatory potentials of given regions of dinosaur anatomy. Feathers are discussed in another review from this issue [19]. Here, we focus on vascular and skeletal information. Few studies have assessed thermoregulation in the postcranial region of dinosaurs. These thermoregulatory studies assessed novel anatomical structures such as plates and sails [40,41]. Most dinosaur thermoregulatory research focuses on cerebral thermoregulation, as neural tissue is more sensitive to temperature fluctuations than other regions of the body [42,43].

Cephalic sites of thermal exchange are regions where blood vessels have close contact with the environment, allowing transfer of heat energy [7,12,43]. Vascular foramina and canal size correlate with the relative importance of these sites for thermal exchange [7]. Comparative vascular distribution in extant taxa revealed that small-bodied dinosaurs had a balanced distribution of cephalic vasculature to all sites of thermal exchange. In contrast, large-bodied dinosaurs exhibited enhancements to one or more sites at the expense of others [7]. Sauropods emphasized nasal and oral pathways, whereas ankylosaurs reduced oral vasculature in favour of enlarging their nasal vasculature [7]. Nasal passage enlargement correlates with large (multi-tonne) body size for most dinosaurs. The intimate relationship between this area and the brain suggests that nasal passages became emphasized for cephalic thermoregulation in large dinosaurs [5].

Initial studies of non-avian dinosaur nasal passages looked for evidence of respiratory turbinates. These folded mucosal structures increase nasal surface area, improving heat exchange efficiency [44–47]. Their near-universal presence in obligate endotherms made turbinates tempting proxies for thermophysiology [46–48]. Turbinate fragility makes fossilization rare. However, the space that respiratory turbinates occupy is substantial. Small airways in dinosaurs led researchers to infer low basal metabolic rates. Later studies using wider taxonomic samples challenged this inference due to a lack of thermophysiological consistency (e.g. whales and pelicans lack turbinates [49], whereas bradymetabolic crocodylians and lizards appear to have respiratory turbinates [4,50]). Further challenging the turbinate inference is the difficulty in accurately measuring the soft-tissue extent of the nasal passage for most dinosaurs, as very few species encase their entire nasal airway in bone.

**Figure 1.**
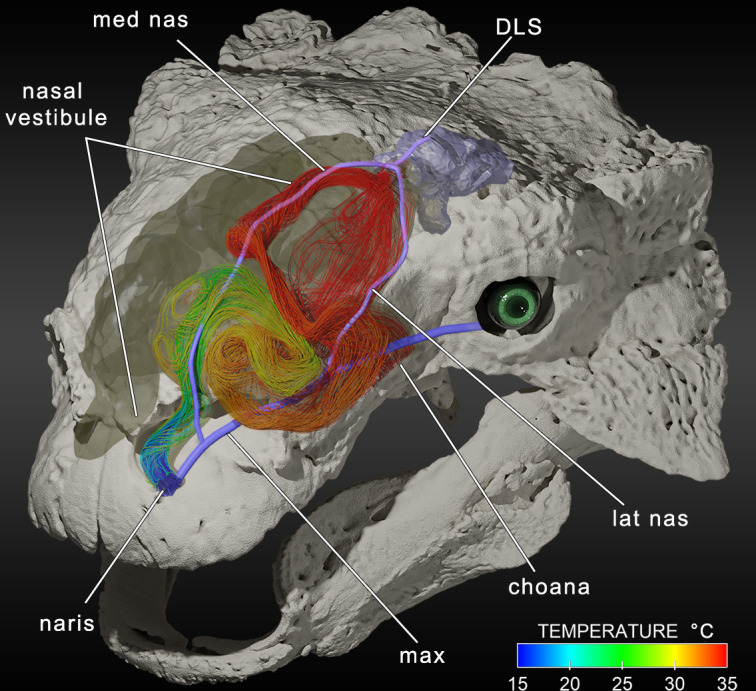
CFD-simulated airflow during inhalation through the left nasal passage and surrounding venous pathway in *Euoplocephalus* (AMNH 5405). DLS, dorsal longitudinal sinus; lat nas, lateral nasal vein; max, maxillary vein; med nas, medial nasal vein. Image created from data from [5].

CT-based reconstructions of ankylosaur skulls revealed well-defined, tortuous nasal passages (figure 1) [51]. Nasal blood vessel reconstructions found that substantial blood flow was present around the nose. These blood vessels were connected to the brain in life (figure 1) [5,7,51]. Computational fluid dynamic (CFD) simulations were used to quantify airflow and heat exchange in nasal passages of two ankylosaurs [5]. Simulated inhalation revealed that inhaled air at 15°C was effectively elevated to an estimated body temperature of 35°C before entering the trachea. Simulated exhalation revealed similar drops in air temperature as air left the nares. Estimated heat and water savings from this counter-current heat exchanger fell within the range of extant animals, despite these dinosaurs lacking respiratory turbinates [5]. Instead of using turbinates to divide the airway into a series of parallel tubes, ankylosaurs retained a single air channel and greatly increased its length (figure 1). A survey of several large-bodied dinosaur groups (e.g. sauropods and several ornithischians [5]) found that this ‘serial’ arrangement appears to have been common. The nasal anatomy of these non-avian dinosaurs is similar to the nasal passages of crocodylians and some lizards such as mastigures (*Uromastyx*) and monitor lizards (*Varanus*) [5,52] albeit to a greater extent in these multi-tonne dinosaurs.

Serially arranged nasal passages require large tidal volumes and relatively slow breathing rates for air exchange in the lungs. This would be challenging for the bellows-like lungs of mammals, but the likely unidirectional airflow in dinosaur lungs (see below) would have made this increased nasal passage length trivial, as also seen in the convoluted tracheae of many birds [53].

In contrast to ankylosaurs, non-avian theropods of all sizes retained a balanced vascular supply to the head and showed no evidence of nasal passage expansion. Non-avian theropods appear to have relied on alternate sources of cephalic heat exchange such as the antorbital sinus [7] and the frontoparietal fossa [8]. The latter is a depressed region within the dorsotemporal fenestra previously interpreted as a muscle attachment site [8]. A comparative soft-tissue and osteological correlate study in extant archosaurs discovered that the frontoparietal fossa housed a complex vascular net or rete, making it a unique site for thermal exchange [8].

## 4. Respiratory system

Due to the direct proximate relationship between the respiratory system and adjacent skeletal tissues, the lower respiratory system has been a popular organ system for dinosaur soft-tissue reconstruction [14,15,54–58]. Postcranial skeletal pneumaticity (PSP) has fuelled decades of interest and speculation [13,54–56,59–64]. Saurischian reconstructions are commonly described as ‘avian style’, preferring the avian lung system as a model based on interpretations of pneumatic foramina in saurischian bones (e.g. [13,54,56,61,62,65,66]). The alternate hypothesis of a crocodylian-like hepatic piston [67] has fallen out of favour as this ventilatory mechanism appears highly derived [68] and is functionally linked to their specialized postcranial skeletal morphology as described in live American alligators (*Alligator mississippiensis*) via ultrasound [69]. Ornithischians may have had another method of ventilation [70], but with a derived morphology and no direct extant relatives, it is difficult to draw robust conclusions.

Forked ribs incising the dorsal surface of adjacent pulmonary tissues have been used as an osteological correlate for a dorsally immobilized gas-exchanging lung in dinosaurs [14,15] and was quantitatively validated via geometric morphometrics [57]. This feature would have provided the skeletal infrastructure for dorsal immobility of pulmonary tissues, a prerequisite for thinning the blood–gas barrier (BGB), and possibly facilitating the survival of early dinosaurs during the hypoxic atmosphere of the Late Triassic [15,57].

PSP is one of the most widely used osteological correlates for reconstructing respiratory soft tissues in ornithodirans, having been well described in saurischians and pterosaurs, indicating the presence of heterogeneous pulmonary structures with invasive pneumatizing diverticula (e.g. [13,55,56,60–63,65,66,71–78]). Pneumatized bones have distinctive pneumatic foramina that create channels where pneumatizing diverticula from pulmonary tissues pass through, creating hollow spaces. Based upon a proposed map of pulmonary structures that invade very specific regions of the postcranial skeleton [54,55], unambiguous correlates of PSP have been used to map cranial and caudal ‘clusters’ of air sac homologues on either side of a gas-exchanging lung in saurischians and pterosaurs derived from a simplified avian *bauplan* (e.g., [54,55,65,79]).

## 5. Cardiovascular system

Researchers inferred the presence of a four-chambered heart in non-avian dinosaurs [80,81] based on the shared, superficial cardiac anatomy of birds and crocodylians [82–84]. A four-chambered heart was likely present in non-avian dinosaurs, but detailed anatomical differences between avian and crocodylian hearts limit interpretations of specific anatomy [85–87]. Physiological reconstructions of non-avian dinosaur hearts have relied on physical constraints that transcend phylogeny to guide heart reconstructions. For example, large dinosaurs (≥1 tonne) would have required completely divided ventricles [88]. This allows the heart to simultaneously pump blood at two vastly different pressures, ensuring perfusion to the body without producing pulmonary oedema [80,81,88–90].

Most dinosaur cardiovascular studies focus on the haemodynamically challenging anatomy of sauropods. How sauropods pumped blood to a head up to 10 m away from their heart remains undetermined [91], with several studies relying on phylogenetically distant giraffes as an extant analogue. Giraffe physiology offers key insights into how extant mammals solve this haemodynamic problem. Yet even in these well-studied animals, there are aspects of giraffe cardiovascular physiology that remain unresolved [92–97]. When extant analogues have reached their explanatory limit, researchers turned to mathematical and computational models to further explore cardiovascular reconstructions. Applying these models to sauropods supports the reconstruction of thick ventricles, stiffened blood vessels and the potential (albeit controversial) presence of cardiovascular siphons [81,98–101]. Researchers looking beyond cardiac anatomy have considered structural adjustments such as neck postures that rarely raised the head 30° above horizontal [91,102], and the unlikely return to a semi-aquatic lifestyle [103]. Sauropod neck posture appears very clade dependent, with at least some clades show anatomical support for high browsing [104–106].

Distal sites from the heart offer further insight into the cardiovascular system of non-avian dinosaurs. The size of long-bone nutrient foramina provides evidence for maximal blood flow rate to those bones, which may be used as a proxy for maximal metabolic rate (MMR) [6]. Applying this method to non-avian dinosaurs, researchers interpreted all their tested species as ‘highly active’ [6,103]. This interpretation carries the caveat that all dinosaur specimens evaluated were substantially larger than the extant dataset used to calibrate the model. Large body size requires larger nutrient foramina, which can lead to misinterpretations of higher activity levels as discussed with ground sloths [107].

Similar to nutrient foramina, cell lacunae in fossilized bones have been used to determine the upper limit of red blood cell (RBC) size [108]. Smaller RBCs reduce the diffusion distance between oxygen and the target tissue, which may be used to assess activity levels of extinct animals. Initial studies applied to dinosauromorphs found relatively high perfusion indicative of high activity levels for this group. However, this increased blood perfusion may also reflect the hypoxic conditions of the Triassic Period [108].

Adjacently related, mathematical models of the aerobic power required for movement in multi-tonne dinosaurs indicate that a high MMR was needed for anything substantially aerobic, including a slow walk [109–111]. Although calculated values exceed the capacity of bradymetabolic animals based on pooled equations, they may not exceed the species variance in MMR observed from empirical studies (e.g. [112,113])

## 6. Digestive system

Rare preservation of the gastrointestinal tract limits our understanding of this area of dinosaur physiology. Multiple proxies have been used to infer digestive physiology in non-avian dinosaurs, including gastroliths, coprolites and tooth morphology. The association of gastroliths with some dinosaur skeletons suggests a partitioned stomach (gizzard) in some species. Gizzards in dinosaurs have been supported based on previous extant phylogenetic bracketing (EPB) studies [114]. However, detailed anatomical comparisons demonstrate gizzards to be variably present in birds [115], with granivorous species having well-developed gizzards [115] and carnivorous species the least-developed gizzards [14]. Gizzards are also observed in crocodylians [116]. However, detailed anatomical comparisons do not support homology with avian gizzards [117]. Instead, archosaurs appear predisposed to stomach partitioning [116], making inferences of gizzards in dinosaurs more dependent on the hypothesized diet, oral anatomy and presence of true gastroliths [118]. Currently, gastroliths have only been found with herbivorous or omnivorous dinosaurs [119–126].

Coprolites and gastric pellets provide direct information on dinosaur diet. Gastric pellets similar to those regurgitated by extant raptors were found with a specimen of *Anchiornis* [16]. These pellets contained multiple disarticulated lizard skeletons, indicating an inability to digest this material and means for anti-peristalsis in paravian theropods. Coprolites attributed to *Tyrannosaurus* contained bone shards, supporting the biomechanical hypothesis that *Tyrannosaurus* was capable of osteophagy [127,128]. Tooth shape, wear and associated jaw biomechanics provide some of the best direct evidence for diet type. These studies have revealed ontogenetic diet shifts and ecological partitioning among dinosaurs [129–132], the macroecology of which is further explored in a separate contribution to this volume [133].

## 7. Attempting validation with extant taxa

### (a) Thermophysiology

**Figure 2.**
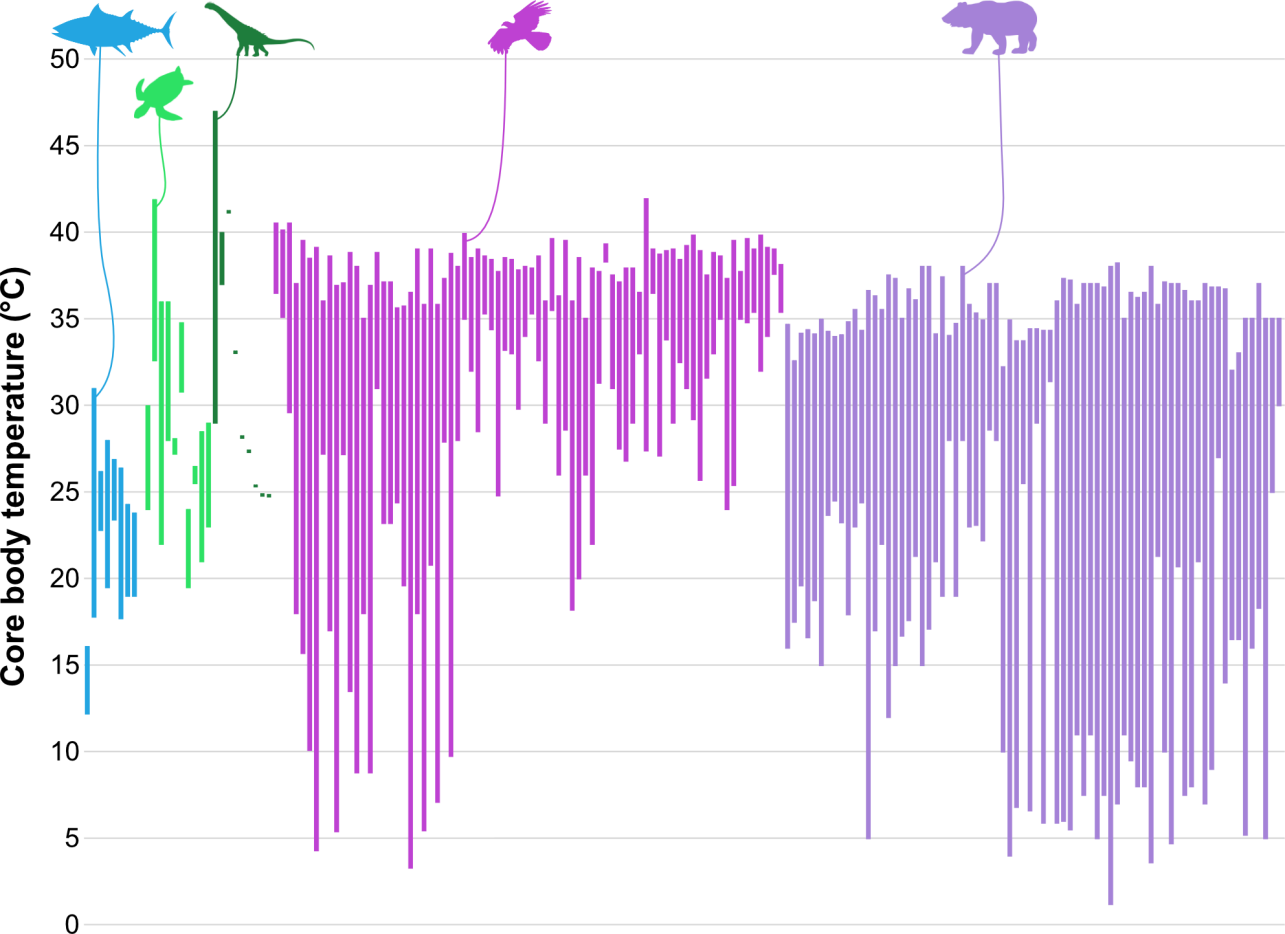
Vertebrate core body temperature ranges (°C). Data are compiled from [35,37,134,137,143–155] and are available in the electronic supplementary material. Blue fish: elasmobranch and fish, light green sea turtle: non-dinosaurian sauropsids, dark green sauropod: non-avian dinosaur estimates, magenta falcon: birds, purple bear: mammals.

‘Endothermy’ in palaeophysiological studies is often used as shorthand for the obligate endothermy of mammals and birds, with little consideration for the myriad cases of heterothermy [134–136] or facultative endothermy [137–142] in extant animals (figure 2). Thermoregulatory diversity [134–136,156–160] and the multiple convergent evolutionary events of facultative endothermy [137–142,161–163] limit the ability of an EPB approach [164,165] for reconstructing dinosaur physiology. Thermophysiology is a dynamic, fluctuating system of processes that mostly occurs at the cellular level over time. These processes rarely preserve in fossils, which only capture a single snapshot from an individual’s life, requiring proxies to reconstruct the extinct animal’s metabolism.

Broader taxonomic skeletochronologic studies of extant animals find no clear distinction between ‘ectothermic bone’ and ‘endothermic bone’ [89,166–168]. Bone appositional growth varies by species, individual and even within the skeleton [169,170]. LAGs, once thought to be a hallmark of bradymetabolic ectotherms, have since been found in several obligate endotherms [171–174]. The large variability in bone formation limits the utility of skeletochronology as a barometer for thermophysiology.

Tests of clumped isotope palaeothermometry with extant animals found that body temperature estimates were within approximately 1°C of the average core temperature of the animal [11,175]. Notably, core temperatures calculated from extant sauropsid eggshells varied between individuals and departed from the expected ranges obtained from the literature [37]. These results demonstrate how intraspecific variation can exceed the variation between broad categories like endotherm and ectotherm (figure 2).

Using ALE signals as proxies for metabolism is enticing, but it is not problem-free. Initial studies calculated RMR based on body mass estimates, which are notoriously difficult to obtain for extinct animals [176]. Further complicating matters, monitor lizards plot as obligate endotherms using this method [10]. Raw data from the original study reveal similar discordance with the low RMR *Tachyglossus*, recovered as ‘more endothermic’ than bats and passerines ([10], electronic supplementary material). Rather than determining whole-body metabolism, ALEs may instead be capturing activity levels or possible MMR for a given animal ([177], see [178] for response). This methodology is still new and will require future studies to tease apart what aspects of metabolism ALEs preserve.

The ability to determine the metabolic rate of non-avian dinosaurs remains a formidable challenge in part due to assumptions of the limitations or freedoms associated with different thermophysiological strategies [179,180] and coupled with conflicting models for the evolution of endothermy [180–183]. Several methods for determining dinosaur metabolism estimate just a single core body temperature and use this as a proxy for metabolic rate. As discussed above, there is extensive overlap in core body temperatures between extant ectotherms and endotherms (figure 2), and these values vary throughout the day and between seasons. There is no static metabolic value, and the dynamic up- and downregulation of metabolic rate makes singular estimates largely uninformative. For example, core body temperature measurements of 31°C have been recorded for both extant ectotherms and endotherms (see figure 2) and require knowledge of the ambient environmental temperature for metabolic interpretation. Using previously described palaeophysiological methods, this singular measurement would lump both groups under the same thermophysiology. Dinosaur palaeontology now has the capacity to estimate body temperature within a few degrees, but these estimates remain limited to single points in time (e.g. bone, enamel or eggshell deposition). These approaches do not capture the complexity of the thermophysiological processes at work, telling us little about whole-body metabolism. Instead, more focused studies on specific aspects of physiology have the greatest potential to inform us on non-avian dinosaur behaviour and life history.

### (b) Thermoregulation

Though nasal passage function in thermoregulation has sparked great interest in palaeophysiology, the nose only minimally alters body temperature. The nasal passages are a relatively small space, and the volumetric flow rate of large animal respiration can only exchange a relatively small amount of heat from the body core. Experimental data on the nasal heat exchange system have shown that it functions mostly in maintaining cerebral homeostasis during normal respiration with a switch to oral heat exchange via panting during periods of heat stress [43,184,185]. Respiratory turbinates present one method for maintaining cerebral homeostasis, but it is not the only way. Birds may have evolved respiratory turbinates in response to the evolution of their enlarged eyes and brains [186], potentially requiring a more immediate heat sink for cerebral thermal homeostasis, such as the avian ophthalmic rete [187].

### (c) Respiration

The avian respiratory system has been used as a guide for reconstructing dinosaur respiration, and advances in bird lung knowledge can help advance reconstructions in dinosaurs. Unique among extant taxa, the avian lower respiratory system is functionally decoupled into an immobilized *gas-exchanging lung* and a flexible set of generally nine *ventilatory air sacs*, dilations of the bronchial tree that serve as ventilatory bellows, pushing/guiding air unidirectionally through the gas-exchanging lung [188–193]. *Non-ventilatory diverticula* variably emerge from the gas-exchanging lung or the ventilatory air sacs [194,195] and extend throughout the coelomic cavity, around joints and subcutaneously. These serve a variety of largely unexplored functions, including enhancing the mechanical advantage of the pectoralis in soaring birds [196] and pneumatizing the postcranial skeleton [72,197]. Unlike mammals, the avian gas-exchanging lung is isovolumetric, locked in place by ribs dorsally and the horizontal septum ventrally [198]. This particular anatomy facilitates an extreme reduction in size of the exchange tissue, thinning of the BGB, and decoupling gas exchange from the ventilatory regions of the respiratory system [199]. This structure facilitates diffusion of oxygen over the respiratory epithelia under hypoxic atmospheric conditions, including flying at high altitudes [200,201].

When those components of the fully avian lung evolved in ornithodirans remains an open question. The ventral boundary of the avian immobilized gas-exchanging lung is maintained by the horizontal septum through attachments to the medial aspects of the sternal ribs via the costopulmonales muscles [198], providing further opportunities for unexplored osteological correlates in dinosaurs and potentially tracking the evolution of the avian lung [202]. If non-avian dinosaurs did not have these structures, they could not have had an ‘avian-style’ lung.

Recent discoveries of unidirectional airflow patterns in non-avian sauropsid lungs suggest that these flow patterns are ancestral for Sauropsida [203–207], and therefore a parsimonious possibility for dinosaurs. Unidirectional airflow patterns of the avian bronchial tree are maintained via aerodynamic valving coupled with the arrangement, angulation and shape of airways, which was determined through rigorous experimental work on ducks and geese (e.g. [188,190,208–210], for reviews see [202,211]). However, sauropsid lungs are morphologically divergent across groups; thus, the ancestral valving mechanism underpinning airflow patterns remains unknown.

The extremely wide range of bronchial morphologies in extant animals makes any specific extinct bronchial tree anatomy or physiological reconstructions nearly impossible. Biological correlates need to be linked to specific causes for effective use of the EPB. Unidirectional airflow may have evolved to enhance crypsis, conserve water, reduce ventilation costs and survival under hypoxic conditions [150,153]. This contrasts with the historical view that specialized avian lungs evolved to facilitate the elevated metabolic demands of flapping flight [212]. Care must be taken not to insert into a dinosaur the derived physiological states of birds or crocodylians when the interpretation of these data remains ambiguous.

**Figure 3.**
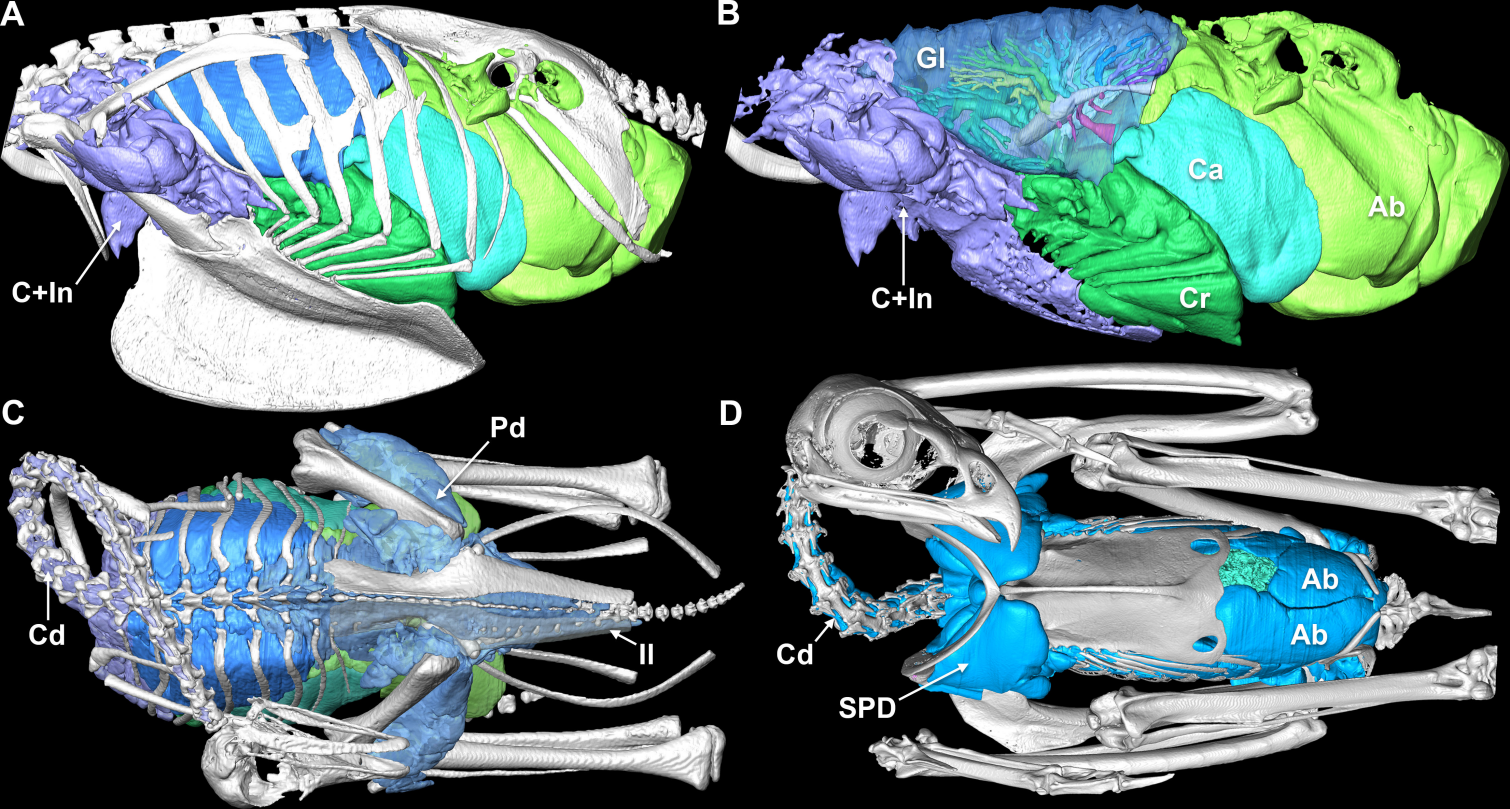
Three-dimensional surface models of the avian respiratory system. Left lateral view of the skeleton (*a*) and respiratory system (*b*) of a naturally deceased and artificially inflated adult African grey parrot (*Psittacus erithacus*), created from [214]. (*c*) Dorsal view of a juvenile artificially inflated common ostrich (*Struthio camelus*; O7), semi-translucent left ilium demonstrates the pneumatized pelvis, data obtained from [195]. (*d*) Ventral view of an adult male artificially inflated red-tailed hawk (*Buteo jamaicensis*) showing non-ventilatory diverticula; created from [196]. Ab, abdominal air sac; C, cervical air sac; Ca, caudal thoracic air sac; Cr, cranial thoracic air sac; Cd, cervical diverticula; Gl, gas-exchanging lung; Il, ilium; In, interclavicular air sac; Pd, pelvic diverticulum. Images not to scale.

The precise developmental mechanism that facilitates PSP invasion remains unknown in birds. Avian skeletal pneumatization begins approximately two months after hatching [213]. Historically, latex injections have been used to study the tissue and negative spaces, because air sacs and pneumatizing diverticula are extremely thin, making this the only method to visualize these tissues (e.g. [72,194]). Recent innovations in computed and micro-computed tomography (CT/µCT) have allowed visualization and quantification of PSP in extinct and extant taxa (figure 3) [59,195,215–218].

A few critical factors confound interpretations of PSP and a fully ‘avian-style’ lung in dinosaurs (see [197] for a full review). (i) A respiratory model of a juvenile common ostrich (*Struthio camelus,* figure 3*c*) indicates that its entire postcranial skeleton is pneumatized by diverticula emerging from the gas-exchanging lung, not ventilatory air sacs [195]. (ii) The function of PSP in extant birds remains an open question. Many avian taxa have secondarily lost PSP for biomechanical reasons [72], but a full survey of Aves has not been completed, nor has any rigorous interspecific experimental work been conducted. (iii) There is no clear evidence of a completely functionally decoupled gas-exchanging lung in a dinosaur, which relies upon clear identification of an osteological correlate for the horizontal septum [202]. (iv) The presence of PSP derived from the swim bladder in some osteoglossiform fish [219–223] suggests additional uses for PSP outside of functions based solely on studies of birds. The swim bladder may be invading foramina that already exist [223] and have a different function from that of birds, but this system is largely unstudied. Importantly, a form of PSP occurring in an extant system outside of Ornithodira—whether homologous or analogous—suggests that pneumatization may have many divergent evolutionary drivers. Collectively, these questions indicate a clear need for further investigation of PSP in extant birds, the pulmonary tissues involved, and the impact of PSP on bone. Addressing these questions will facilitate more rigorous reconstructions of ornithodiran pulmonary biology.

## 8. Recommendations

The neontological literature is rife with examples of extensive intraspecific variation [214,224–229]. Dinosaur palaeontology acknowledges individual variation when reconstructing muscles, ontogenetic growth and sexual dimorphism [230–233], but rarely considers it for palaeophysiology. Soft-tissue reconstructions are often based on a few individuals from extant exemplar species (e.g. *A. mississippiensis* or *Gallus gallus*). Sauropsida is the second-most speciose group of vertebrates today with over 11 000 species of extant birds [234] and over 12 000 species of extant reptiles, including 27 crocodylians [235]. The depth of intra-/interspecific variability remains largely unexplored. This large knowledge gap limits EPB-based inferences. A condition of this method is that a trait and its functional link are validated across the extant clades of interest [164,165]. As presented in the examples above, many traits once thought to be directly linked to metabolic physiology quickly fall apart as a wider range of species are examined. Broad categories, such as core body temperature, are not informative proxies for determining metabolism due to the large overlap between extant ectotherms and endotherms. Difficult to fossilize physiological or behavioural variables, such as hibernation and torpor, also confound proxies. As with growth rate studies, inter-/intraspecific variation is necessary to accurately reconstruct an extinct animal’s physiology.

To produce a solid palaeophysiological reconstruction, we recommend collecting extant data from a minimum of three to five individuals each across several species. This sample size will account for at least some normal variation [69,206,236,237]. Larger sample sizes increase robustness of statistical analyses, but considerations should be made for cost, time and avoiding unnecessary waste of animal life [237–239]. Efforts to account for intraspecific variability in relevant biological factors (e.g. body size, sex, ontogenetic stage, reproductive and migratory status) are needed to verify that a chosen specimen is not an aberrant individual. Rare species offer unique insights into physiological potential, even when the sample size of that species is only one. We encourage the use of rare species as long as authors note their sample size limitations. The use of captive-bred specimens can introduce confounding artefacts [240,241]. As this is difficult to avoid (e.g. bird species that are not easily accessed in the wild), we recommend explicitly stating any potential artefacts of captive-breeding, like obesity, metabolic bone disease or arthritis, that may result in different physiological outcomes compared to their wild counterparts.

When using imaging to reconstruct anatomy or physiology, reconstructions should be validated against physical specimens any time a unique observation is recorded. For instance, if an avian CT scan reveals a unique air sac–muscle relationship, that specimen or similar ones should be dissected to confirm the radiographic observation [196]. Soft-tissue scans of deceased individuals should be performed on healthy, recently deceased specimens whenever possible [197,240], especially when reconstructing lung and brain material. Palaeontologists must often rely on ‘salvaged’ specimens for their studies, and time of death for these specimens is rarely known. Pulmonary tissue starts decaying within hours after death, and repeated freeze–thaw cycles can result in fluid build-up in the lung and adjacent pneumatized spaces of birds, confounding results [240]. Collaborating with wildlife rehabilitation centres or zoological medicine veterinary clinics offer a way to collect specimens frozen immediately after death and before decomposition starts. This vastly improves the likelihood of retaining pristine anatomy and obtaining the most accurate information.

Palaeophysiological inferences should be functionally and experimentally validated using live-animal experiments whenever possible (e.g. [8,69,241]). Computational models of extinct physiology must first be validated against extant animals, and the limitations/assumptions of those models should be explicitly discussed when interpreting the data. Using equivalent analyses on more constrained groups (taxonomically and spatially) would also strengthen analyses. We encourage researchers to clarify the limitations of their results and to avoid extravagant, headline-catching claims. Technology has enabled palaeontology to make huge strides in our understanding of dinosaur physiology. The allure of powerful new methods for interpreting palaeophysiology can overshadow its limitations. As long as researchers remain transparent regarding the limitations related to constructing and validating their hypotheses, the field of palaeophysiology will continue to advance.

## Data Availability

Dinosaur fossil occurrences are provided in the electronic supplementary material, citing Paleobiology Database contributors and literature. Figure 1 is adapted from [5]. Temperature range data for figure 2 are provided in the electronic supplementary material, citing literature containing the data points. Data for figure 3 are available from the Dryad Digital Repository [242] and the parrot from MorphoSource (doi:10.17602/M2/M553437). The ostrich data are associated with [195] and the parrot data with [214]. Supplementary material is available online [243].
